# LPS-Challenged TNF*α* Production, Prostaglandin Secretion, and TNF*α*/TNFRs Expression in the Endometrium of Domestic Cats in Estrus or Diestrus, and in Cats with Pyometra or Receiving Medroxyprogesterone Acetate

**DOI:** 10.1155/2014/689280

**Published:** 2014-06-15

**Authors:** Ewelina Jursza, Anna Z. Szóstek, Mariusz P. Kowalewski, Alois Boos, Kiyoshi Okuda, Marta J. Siemieniuch

**Affiliations:** ^1^Department of Reproductive Immunology and Pathology, Institute of Animal Reproduction and Food Research of the Polish Academy of Sciences, Tuwima Street, 10-748 Olsztyn, Poland; ^2^Institute of Veterinary Anatomy, Vetsuisse Faculty, University of Zurich, Winterthurerstrasse 260, 8057 Zurich, Switzerland; ^3^Graduate School of Natural Science and Technology, Okayama University, Tsushima Naka, Okayama 700-8530, Japan

## Abstract

Progesterone (P_4_) derivatives which are commonly used to block the cyclicity of domestic cats disturb the endocrine balance in the endometrium. The aims of this study were (i) to examine whether lipopolysaccharide (LPS) is responsible for enhancement of tumor necrosis factor-*α* (TNF*α*) secretion by the feline endometrial epithelial and stromal cells *in vitro*, (ii) to know whether immunolocalization of TNF*α*/TNFR1 and TNFR2 differs in cats at estrus or diestrus, receiving medroxyprogesterone acetate and suffering from pyometra, and (iii) to determine if TNF*α*-challenged prostaglandin secretion is stopped by prostaglandin synthases inhibitors. A total of 37 domestic adult cats in estrus or diestrus, receiving octane medroxyprogesterone or having clinical symptoms of pyometra, were enrolled in this study. The results obtained showed a distinct increase in LPS-challenged TNF*α* secretion in endometrial epithelial, but not stromal cells. TNF*α* augmented PG secretion was blocked by phospholipase A_2_ (PLA_2_) and cyclooxygeanase-2 (COX-2), but not by mitogen-activated protein kinase (MAPK) inhibitor. TNF*α*/TNFR1 and 2 protein expressions were limited mostly to the surface and glandular epithelium. TNF*α*/TNFRs protein was upregulated in the inflammatory uterus and hence may be involved in development of pathologic changes in the endometrial glands in cats receiving exogenous P_4_ as a hormonal contraceptive.

## 1. Introduction

Cystic endometrial hyperplasia and pyometra complex is one of the most common and important reproductive disorders in cats [1, 2] and dogs [2, 3]. This syndrome is a sequel to progesterone (P_4_) priming of the endometrium and is mostly caused by an increasing endometrial infection with vaginal bacteria [4]. An endocrine disturbance, particularly with respect to P_4_ imbalance, is prerequisite for development of endometritis-pyometra complex (EPC) [4, 5]. A pyometra is the most severe form of endometrial disease, accompanied by accumulation of purulent fluid in the uterine lumen and general toxemia. Microbial infection is necessary for the development of pyometra; however, bacterial colonization of the endometrium is always a secondary event to the hormonal disturbances. Both female cats and dogs are susceptible to EPC, although in bitches this disorder is more frequent because of prolonged exposure of the endometrium to elevated P_4_ levels during the nonpregnant luteal phase [2]. For a long time, it was believed that ovulation in the queen is induced by multiple copulations, and so, because of the absence of mating, a single cat in a household should be protected from progesterone stimulation of the endometrium. However, in several studies describing EPC in cats, many of the affected animals were reported to live in single-cat households but exhibited corpora lutea, suggesting the occurrence of ovulation without mating in this species [6–8]. Finally, the presence of noncopulatory ovulation in domestic cats was confirmed by Lawler et al. [9]. Even if a nonpregnant luteal phase appears in the cat, it lasts only for 30–35 days [10, 11]. After that time, P_4_ levels drop to a basal value of around 1 ng mL^−1^. The shorter time of endometrial exposure to elevated P_4_ level may explain the lower susceptibility of the feline endometrium to EPC compared with dogs. However, P_4_ derivatives, among them medroxyprogesterone acetate or megestrol acetate, which are commonly given to female cats for silencing sexual behavior, cause hypertrophy of the endometrium, dilatation and hypertrophy of the endometrial glands, or stimulation of the endometrial glands to produce excessive mucus secretion [6, 12]. Because of this, P_4_ derivatives collectively facilitate the development of EPC.

Although the endometrium is usually a sterile environment, microbial flora colonizing the lower urogenital tract of clinically healthy queens may intrude into the uterine lumen, resulting in the development of pyometra under favorable conditions. The most common uterine infections in cats are caused by the Gram-negative bacterium* Escherichia coli *[8]; however, this species, accompanied by* Proteus *spp. or* Enterococcus faecalis*, was also isolated from the vagina of healthy cats [13]. Gram-positive bacteria were present to a lesser extent and were overrepresented by* Streptococcus canis*,* Staphylococcus aureus*, and* Staphylococcus epidermidis* in queens without any clinical signs of EPC (for review see Clemetson and Ward [14]). The mucosal membranes, including the endometrium, are involved in host defense against pathogens. The epithelial or stromal cells localized in the mucosa use pattern-recognition receptors to detect the presence of pathogens by recognizing pathogen-associated molecular patterns (PAMP), such as microbial components, including lipopolysaccharide (LPS) or lipoteichoic acid and lipoproteins. Pathogen-associated molecular patterns are recognized by toll like receptors (TLRs) [15]. The localization of TLRs in the endometrium of cats receiving medroxyprogesterone acetate or those suffering from pyometra or cats in estrus or diestrus is now under study (Jursza and Siemieniuch, unpublished, 2014). Activation of TLRs affects the secretion of cytokines, among them tumor necrosis factor *α* (TNF*α*) and chemokines [16]. In the human endometrium, TNF*α* is synthesized by immune-competent cells, such as macrophages or monocytes, as well as by endometrial fibroblasts [17] and glandular epithelial cells [18]. We showed recently that TNF*α* is produced in the feline endometrium in a cycle-dependent manner and is responsible for augmentation of prostaglandin secretion [19]. Otto and Rawlings [20] observed that supernatants of peritoneal exudate cells from cats produced vast amounts of TNF*α* when exposed to LPS. However, data concerning the role of the feline endometrium in innate responses during bacterial contamination remain sparse.

In the present study, we hypothesized that LPS induces TNF*α* secretion in the endometrial epithelial and stromal cells of the domestic cat* in vitro* and TNF*α* affects prostaglandin (PG) synthesis in endometrial cells. To address this hypothesis, the following topics were examined: (i) LPS-challenged secretion of TNF*α* by cultured epithelial or stromal endometrial cells, (ii) spatial and temporal localization of TNF*α* and its receptors in the feline endometrium, and (iii) abolition of TNF*α*-challenged prostaglandin secretion by several PG synthase inhibitors.

## 2. Materials and Methods

### 2.1. Animals and Collection of Uteri

All procedures were approved by the Local Animal Care and Use Committee in Olsztyn, Poland (number 60/2010/DTN). A total of 37 mature, domestic shorthair cats were enrolled in this study. The cumulative information provided by inspection of the ovaries at ovariohysterectomy (OHE), circulating levels of P_4_, and, when available, information from the owner were used to stage the estrous cycle of each animal. No pharmacological treatment was performed to provoke ovulation in the animals. Queens were checked daily for behavioral signs of estrus (treading of the hind feet, lordosis, and tail deflection). The uteri were assigned to (1) estrus (E) group (*n* = 19); (2) diestrus (D) group (*n* = 8); (3) hormonally treated with medroxyprogesterone acetate (MPA, Depo-Promone, Pfizer Animal Health, Louvain-la-Neuve, France) group (*n* = 7), in which animals received an injection of 50 mg MPA every 4 months and were ovariohysterectomized between four to twelve months from the first injection; and (4) pyometric (PYO) group (*n* = 3).

Tissues were washed immediately after surgery with sterile saline to remove blood contamination, placed into fresh sterile saline at 4°C, and transported to the laboratory within 1 h. Uterine horns were slit longitudinally and pieces of endometrium were prepared and washed in a fresh saline. One piece of an endometrium obtained from E (*n* = 4), D (*n* = 8), MPA (*n* = 7), or PYO (*n* = 3) was formalin-fixed and used for further immunohistochemical studies. The rest of the endometrial pieces obtained from cats in estrus or diestrus were used for cell isolation.

*Experiment 1 (in vitro endometrial cell experiments)*

#### 2.1.1. Isolation and Culture of Feline Endometrial Cells

The present protocol for isolation of endometrial epithelial or stromal cells contains some important modifications of the one previously described [21]. The major modifications were the use of endometria from females in the estrus phase for isolation of epithelial cells and the use of endometria from diestrus females for isolation of stromal cells. These changes provided better efficacy and lower cross-contamination of the two types of cells. The uterine horns were separated and cut longitudinally and small pieces of endometrium were dissected from the myometrial layer with a scalpel blade and washed once in sterile Hanks' balanced salt solution (HBSS) containing 20 *μ*g mL^−1^ of gentamicin (Invitrogen, San Diego, CA, USA). The endometrial fragments were minced into small pieces (approximately 1 mm^3^) and digested by stirring for 60 min in 50 mL of sterile HBSS containing collagenase (2 mg mL^−1^, Sigma Aldrich, St. Louis, MO), DNase I (200 *μ*g mL^−1^, Sigma Aldrich), and dispase (1.2 U mL^−1^, Sigma Aldrich).

The mixture of cells together with cellular debris was filtered through a pair of metal meshes (100 *μ*m and then 80 *μ*m) to remove undissociated tissue fragments. Then, the endometrial cell suspension was washed three times by centrifugation 10 min at 100 ×g, at 20°C with Dulbecco's modified Eagle's medium (DMEM/Ham F-12 (D/F), 1 : 1 (v/v), Sigma Aldrich) and suspended in 10 mL fresh medium. The cell concentration was counted using a hemocytometer. Cell viability exceeded 90% as assessed by 0.04% (w/v) trypan blue dye exclusion.

The final pellet of isolated endometrial cells was resuspended in D/F culture medium supplemented with 10% (v/v) fetal calf serum (FCS, Sigma Aldrich) and 20 *μ*g/mL of gentamicin (Invitrogen). The cells were seeded at a density of 2 × 10^5^ viable cells/mL in 75 cm^2^ culture flasks (Greiner Bio-One, Monroe, NC) and cultured at 37.5°C in a humidified atmosphere of 5% CO_2_ in air. The medium was changed one hour after plating, by which time selective attachment of stromal cells had occurred. In the case of luteal stage endometria, the amount of stromal cells was enough for plating into two culture flasks at the concentration stated above. The rest of the culture medium, containing a small amount of epithelial cells, was discarded. In the case of estrus stage endometria, the medium was changed one hour after plating; this medium, which contained the epithelial cells but was free of already-attached stromal cells, was placed into a new culture flask. The amount of epithelial cells was enough for plating into one culture flask at the concentration stated above. In case of sporadic contamination of the epithelial cell cultures by stromal cells, 0.025% trypsin (Sigma Aldrich) diluted in sterile Ca^2+^- and Mg^2+^-free phosphate-buffered saline (PBS) was used for 3-4 min at 20°C to detach stromal cells. After reaching confluence (3-4 days after the start of the culture), the cells were rinsed twice with sterile PBS. In order to collect stromal cells, the cell cultures were incubated in 0.025% trypsin and 0.008% ethylene diamine tetraacetate (EDTA) for 4-5 min at 20°C. To collect epithelial cells, the cell cultures were incubated with 0.008% EDTA for 2 min at 20°C. The cell cultures were then rinsed in PBS to remove contaminating stromal cells or fibroblasts. The cells were then incubated with 0.025% trypsin for 8–10 min at 20°C and, at the end of the incubation period, 25 mL of D/F supplemented with 10% FCS was added to stop the enzymatic reaction. Both types of cells were washed once by centrifugation (10 min at 100 ×g). The pellets of both types of cells were resuspended in 10 mL of fresh D/F medium and the cell concentration was counted using a hemocytometer. Cell viability exceeded 90% as assessed by 0.04% (w/v) trypan blue dye exclusion. The cells were seeded at a density of 2 × 10^5^ viable cells/mL in a 48-well cluster dish (Greiner Bio-One, Monroe, NC) and harvested as described for the primary cell cultures for Experiments 1 and 2 or were seeded at a density of 2 × 10^4^ per well in a MultiScreen sterile 96-well plate with a PVDF membrane (Millipore) using fresh D/F without phenol red supplemented with 0.1% BSA for Experiments 1.1, 1.2, 1.3, 1.4, and 1.5.

*Experiment 1.1 (immunofluorescence of endometrial cells)*. Epithelial and stromal cells were identified using immunofluorescent staining for specific markers of epithelial cells (cytokeratin) or stromal cells (vimentin), as described previously [22]. Briefly, the epithelial- or stromal-derived cells were seeded at 2 × 10^5^ cells/mL in special slide flasks (Nunc, Roskilde, Denmark) and cultured. After 48 h of culture, the slides were washed three times in PBS, fixed in methanol for 10 min, and air-dried. Slides were then washed three times in PBS. Triton X 100 0.01% in PBS was added to the cell cultures for 10 min at 20°C. Then, the slides were again washed three times in PBS and incubated for 12 h at 4°C with the primary antibody against either cytokeratin (mouse monoclonal anti-human cytokeratin peptide 18; dilution 1 : 100, Sigma Aldrich) or vimentin (mouse monoclonal anti-pig eye lens vimentin; dilution 1 : 200; Sigma Aldrich) in PBS. Subsequently, the slides were washed three times in PBS and then incubated with the second antibody (anti-mouse IgG conjugated to insert types of fluorescent dyes used) for 1 h at 20°C and protected against light. The controls were prepared as described above in the absence of the primary antibody. Images were captured using a digital camera (Leica, Solms, Germany) and visualized under a fluorescence microscope (Olympus, USA).

*Experiment 1.2 (assessment of cell viability). *Epithelial cells isolated from cats in estrus (*n* = 4) were plated in 96-well dishes at a density of 1 × 10^4^ cells mL^−1^ and incubated for 4 h at 37.5°C with the same treatments as listed in Experiment 1.5. After culture, the cells were trypsinized in order to count the cell numbers which was used to standardize the results. The assay was based on the cleavage of the yellow tetrazolium salt MTT [3-(4,5-dimethyl-2 thiazolyl)-2,5-diphenyl-2H-tetrazolium/Br] to purple formazan crystals by the mitochondria of metabolic active cells. The absorbance was measured at 450 nm using a microplate reader (model 450; Bio Rad, Hercules, CA, USA). Cell viability (%) was calculated as follows: cell viability (%) = 100 × (*A*_test_/*A*_control_), where *A*_control_ is the mean *A* of nontreated wells and *A*_test_ is the mean *A* of all the experimental wells. The standardization of results was based on DNA content [23].

**Figure 1:**
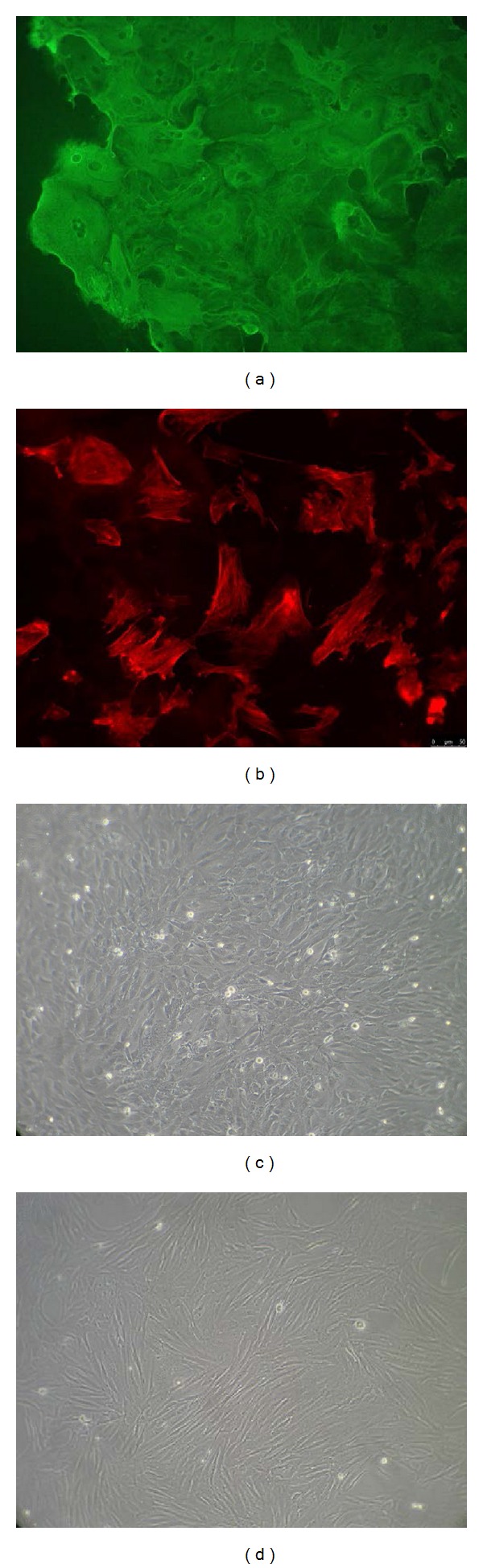
Epithelial and stromal cell culture. Homogeneity of the epithelial (a) and stromal (b) cell cultures was assessed by immunofluorescent staining for specific markers of epithelial cells (cytokeratin) and stromal cells (vimentin). Morphology of cultured feline endometrial cells: primary cultures of epithelial (c) and stromal (d) cells under phase-contrast microscopy, on day 4 of culture.

*Experiment 1.3 (LPS-challenged secretion of TNF*α*)*. To determine TNF*α* production by endometrial cells, the equine enzyme-linked immunosorbent assay (ELISpot) was used (R&D Systems, Minneapolis, USA) following the manufacturer's instructions. Stromal and epithelial cells isolated from cats in estrus (*n* = 4) or diestrus (*n* = 4), derived from passage 1, were seeded at a density of 2 × 10^4^ per well in a MultiScreen sterile 96-well plate with a PVDF membrane (Millipore) using fresh DMEM without phenol red supplemented with 0.1% BSA and antibiotics and antimycotic solution. The density of cell seeding was established in a preliminary experiment. Then, cells were incubated with either vehicle alone, LPS purified from* E. coli* O55:B5 (Sigma) (50 ng/mL), or LPS + TNF*α* (50 ng mL^−1^ + 1 ng mL^−1^) for 24 h. The following controls were used: positive control (TNF*α*); negative control (unstimulated cells at the same density as stimulated cells); background control (culture medium alone); and detection antibody control (PBS substituted for the detection antibody). Spots were visualized using the BCIP/NBT substrate detection system according to the manufacturer's instructions (Figure 1). The spots were analyzed using an Eli. Scan scanner and software (*A.EL.VIS GmbH*; Hannover; Germany).

*Experiment 1.4 (optimization of culture conditions: dose-dependent effects of TNF on P* *GF*_2*α*_ *and P* *GE*_2_ *secretion)*. To validate the cell culture model and to choose an optimal dose of TNF*α* (among 0, 0.1, 1, and 10 ng mL^−1^) on prostaglandin secretion, epithelial endometrial cells isolated from estrus cats were used (*n* = 6). After 4 h incubation, conditioned media from the control and treatment groups were collected and stored at −20°C until PGF_2*α*_ and PGE_2_ analyses. Standardization of the results was based on DNA content [23].

*Experiment 1.5 (effects of TNF, nimesulide, PD, anthranilic acid, and arachidonic acid (AA) on P* *GF*_2*α*_ *and P* *GE*_2_ *secretion from cultured endometrial epithelial cells)*. To study modulation of secretory function by the factors under investigation, epithelial endometrial cells isolated from estrus cats (*n* = 6) were used because the epithelial cells, in contrast to stromal cells, were responsible for distinctive TNF*α* secretion followed by LPS stimulation. After the cells reached 80–90% confluence, they were washed with M199 supplemented with 0.1% BSA and then incubated at 37.5°C in fresh D/F medium supplemented with 0.1% BSA and 20 *μ*g mL^−1^ of gentamicin (Invitrogen, USA). After 30 min stabilization, cells were incubated for 4 h with one of the following treatments: (1) control (without factors); (2) 1 ng mL^−1^ which corresponds to 10^−9^ M TNF*α* (Sigma Aldrich); (3) 10^−6^ M AA (Sigma Aldrich) as a positive control; (4) 10^−8^ M nimesulide (NS-398, Sigma Aldrich) which is a selective COX-2 inhibitor; (5) 10^−8^ M PD 98059 (Calbiochem, Darmstadt, Germany), which is a selective, reversible, and cell-permeable inhibitor of MAP kinase; (6) 10^−8^ M N-(p-amylcinnanoyl)anthranilic acid (ACA 104550) (Calbiochem), which is a cell-permeable inhibitor of phospholipase A2. After 30 min preincubation at 37.5°C, TNF*α* was added at 1 ng mL^−1^ to the wells containing factors (4), (5), and (6). After 4 h incubation at 37.5°C, conditioned media from the negative or positive control and treatment groups were collected and stored at −20°C until PGF_2*α*_ and PGE_2_ analyses. Standardization of the results was based on DNA content [23].

*Experiment 2 (spatial localization of TNF*α*/TNFRs protein in the feline endometrium)*. Formalin-fixed, paraffin-embedded tissues were cut with a microtome (2-3 *μ*m) and mounted on SuperFrost Plus microscope slides (Menzel-Gläser, Braunschweig, Germany). The experimental protocol was as previously described for feline ovaries and placenta [24]. Briefly, slides were deparaffinized and rehydrated in a graded ethanol series and then incubated in citrate buffer (10 nM, pH 6.0) for 15 min under microwave irradiation at 560 W for antigen retrieval. Then, sections were incubated in 0.3% H_2_O_2_ in methanol for 30 min to quench endogenous peroxidase and then washed in IHC-buffer/0.3% Triton X pH 7.2–7.4 (0.8 mM Na_2_HPO_4_, 1.47 mM KH_2_PO_4_, 2.68 mM KCl, and 1.37 mM NaCl). Blocking of nonspecific binding sites was performed with 10% goat serum. The following primary antibodies were used: TNF*α* (rabbit polyclonal to TNF*α*; dilution 1 : 1500, Abcam, Cambridge, MA, USA), TNFR1 antibody (rabbit polyclonal to TNF Receptor I; dilution 1 : 1500, Abcam), and TNFR2 (rabbit polyclonal to TNF Receptor II; dilution 1 : 25, Abcam). An isotype control was done to avoid false positive results. The endometrial sections were incubated with serial dilutions of preimmunized rabbit serum (Vector Laboratories, Burlingame, USA), starting at 1 : 25. No positive staining was observed at a 1 : 25 dilution of preimmunized rabbit serum.

Sections were incubated overnight at 4°C. After washing with IHC-buffer, slides were incubated for 30 min at 20°C with either biotinylated anti-rabbit IgG (secondary antibody; dilution 1 : 100) (Vector Laboratories). For enhancing signals, sections were incubated with the avidin-biotin-peroxidase complex (Vectastain ABC kit, Vector Laboratories) for 30 min at 20°C. After washing with IHC buffer, sections were allowed to react with the substrate diaminobenzidine (DAB; DakoCytomation, Glostrup, Denmark) according to the manufacturer's instructions. Slides were counterstained with hematoxylin, rinsed under running tap water for 5 min, dehydrated in a graded ethanol series, and mounted in mounting medium DPX (Panreac Quimica Sau, Barcelona, Spain).

### 2.2. Immunohistochemical Scoring

A blind assessment of immunolabeling intensity was performed with a Leica (Solms, Germany) microscope. Immunoreactivity was scored taking into account the staining intensity and distribution of specific staining. Ten sight fields were inspected from each slide. Positive signals were indicated as a dark brown to brownish color. The TNF*α*, TNFR1, and TNFR2 immunoexpressions were examined independently at the surface epithelium, epithelial glands, and endometrial stroma. The immunolabeling intensity was scored as negative (0), weak (1), moderate (2), or strong (3).

### 2.3. Hormone Determinations

For PGF_2*α*_ and PGE_2_ measurements, the commercial PGF_2*α*_ high sensitivity EIA kit and the PGE_2 _high sensitivity EIA kit (both from ENZO Life Sciences Inc., Farmingdale, NY, USA) were used and run according to the manufacturer's instructions.

The sensitivity of the PGF_2*α*_ assay was 0.98 pg/mL. The cross-reactivity for various PGs and their metabolites was as follows: PGF_2*α*_: 100%, PGF_1*α*_: 11.82%, PGD_2_: 3.62%, 6-keto-PGF_1*α*_: 1.38%, PGI_2_: 1.25%, and PGE_2_: 0.77%. The inter- and intra-assay variation coefficients were 10.8% and 8.6%, respectively. The sensitivity of the PGE_2_ assay was 8.26 pg/mL. The cross-reactivity for various prostaglandins and their metabolites was as follows: PGE_2_: 100%, PGE_1_: 70%, PGE_3_: 16.3%, PGF_1*α*_: 1.4%, PGF_2*α*_: 0.7%, and 6-keto-PGF_1*α*_: 0.6%. The inter- and intra-assay variation coefficients were 12.2% and 6.9%, respectively.

### 2.4. Statistics

Data concerning concentrations of PGE_2_ or PGF_2*α*_ in conditioned media, epithelial cell viability, and results obtained with ELISpot were analyzed by one-way analysis of variance (ANOVA) and followed by the Newman-Keuls multiple comparison test among means (GraphPad PRISM, version 6.0; GraphPad Software Inc., San Diego, CA, USA). Prostaglandin E_2_ or PGF_2*α*_ concentrations in the incubation media are shown as a mean ± SEM of values obtained in all experiments. Significance was defined as a *P* value of <0.05.

## 3. Results

### 3.1. Cell Culture Characterization

**Figure 2:**
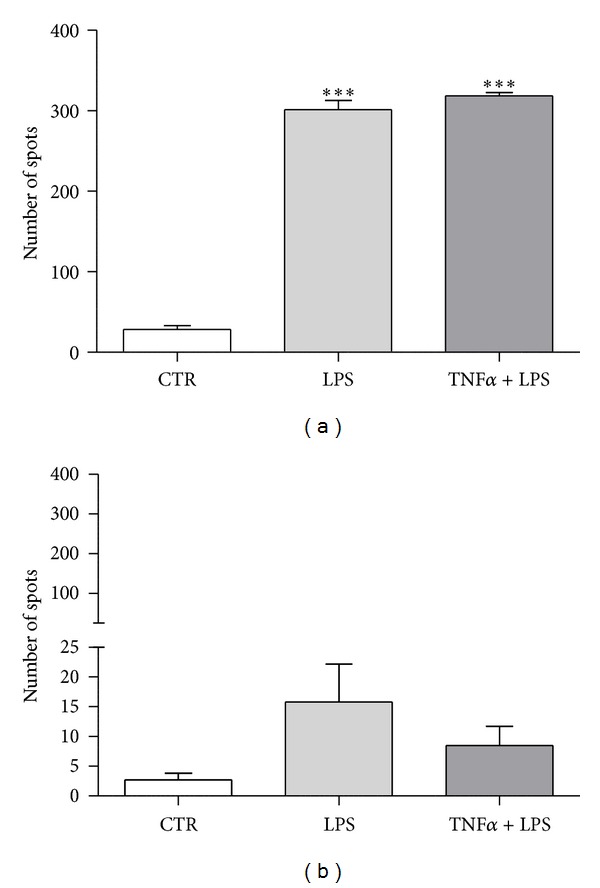
Number of spots after incubation of epithelial cells (a) or stromal cells (b) with LPS at 50 ng mL^−1^ or LPS plus TNF*α* (at 50 ng mL^−1^ and 1 ng mL^−1^, resp.). Asterisks indicate statistical differences between numbers of spots depending on the treatment (∗∗∗*P* < 0.001).

Cultured cells presented two distinct characteristic morphologies: (1) cuboid or cylindrical shape with a distinct round nucleus (Figure 1(c)) and (2) spindle-shaped or elongated with a slightly visible nucleus (Figure 1(d)). Staining with cytokeratin or vimentin allowed both cell types to be distinguished. Cuboid-shaped cells stained with cytokeratin were classified as epithelial cells (Figure 1(a)), whilst cells stained positively with mesenchymal cells marker vimentin were classified as stromal cells (Figure 1(b)).

### 3.2. Assessment of Cell Viability

There were no statistically important changes in cell viability in epithelial cell cultures from cats in estrus (*P* > 0.05) (data not shown).

### 3.3. Quantification of LPS-Challenged TNF*α* Secretion

ELISpot showed that stromal cells produced a small amount of TNF*α* after treatment with LPS (50 ng mL^−1^) and LPS together with TNF*α* (50 ng mL^−1^ plus 1 ng mL^−1^) (Figure 2(a)). In contrast to stromal cells, epithelial cells secreted distinctly greater amounts of this cytokine (*P* < 0.001) (Figure 2(b)). Epithelial cells produced 10.57- or 11.18-fold more spots when stimulated with LPS or LPS plus TNF*α*, respectively, than the control. Comparing the TNF*α* secretion profile in epithelial versus stromal cells, the former secreted 19.07-fold more TNF*α* after challenging with LPS and 37.5-fold more TNF*α* after challenging with LPS plus TNF*α* than stromal cells.

### 3.4. Quantification of Prostaglandins in Culture Media

**Figure 3:**
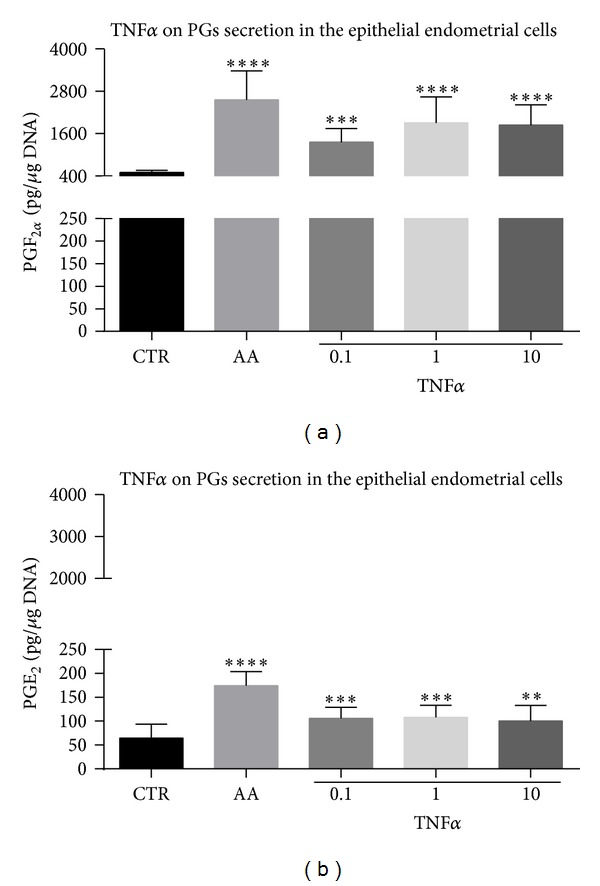
Dose-dependent effect of TNF*α* on PGF_2*α*_ (a) and PGE_2_ (b) secretion/concentrations as determined by immunoassay in endometrial epithelial cells (surface and glandular epithelium). Asterisks indicate statistical differences between prostaglandin (PG) levels depending on the treatment (∗∗*P* < 0.01, ∗∗∗*P* < 0.001, and ∗∗∗∗*P* < 0.0001).

In Experiment 1.4, the accumulated PGF_2*α*_ and PGE_2_ concentrations in conditioned media collected from the endometrial epithelial cell cultures increased at 4 h after TNF*α* treatment at 0.1 (*P* < 0.001), 1 (*P* < 0.0001 or *P* < 0.01 for PGF_2*α*_ and PGE_2_, resp.), or 10 ng/mL (*P* < 0.0001 or *P* < 0.01, resp.) (Figures 3(a) and 3(b), resp.). Arachidonic acid increased both types of PGs secretion in epithelial cells compared with controls (*P* < 0.0001).

**Figure 4:**
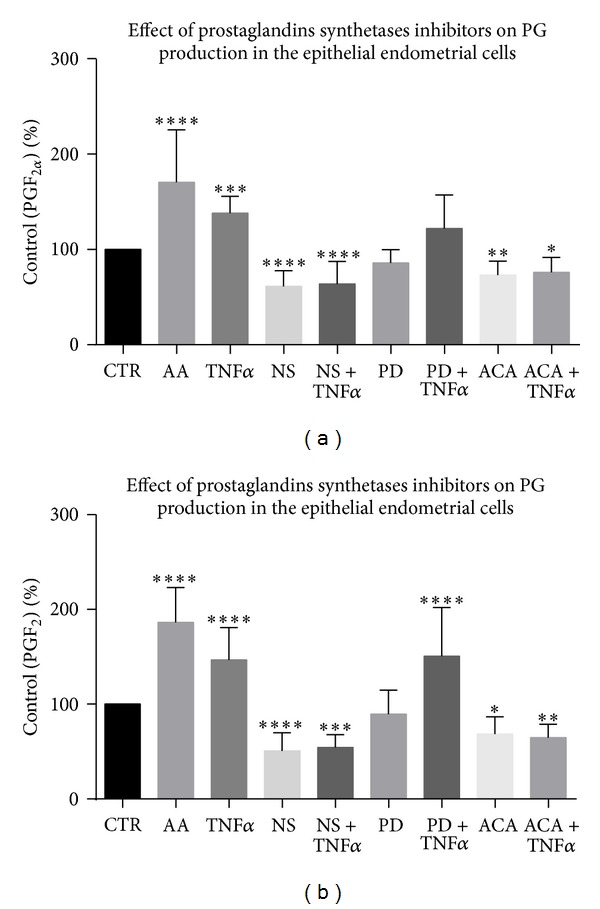
The content of PGF_2*α*_ or PGE_2_ after different treatments as determined by immunoassays. Asterisks indicate statistical differences between prostaglandin levels depending on the treatment (∗*P* < 0.05, ∗∗*P* < 0.01, ∗∗∗*P* < 0.001, and ∗∗∗∗*P* < 0.0001).

In Experiment 1.5, PG production by feline epithelial endometrial cells was examined. Prostaglandin F_2*α*_ production was increased after stimulation with TNF*α* and AA (*P* < 0.001 and *P* < 0.0001, resp.) and decreased after stimulation with NS and NS/TNF*α* (*P* < 0.001), with ACA (*P* < 0.01), and with ACA/TNF*α* (*P* < 0.05). An inhibitor of MAP kinase, PD, alone or together with TNF*α* did not affect PGF_2*α*_ secretion in endometrial epithelial cells (Figure 4(a)).

Prostaglandin E_2_ production increased after stimulation with TNF*α* and AA or after simultaneous treatment with PD and TNF*α* (*P* < 0.0001). Prostaglandin E_2_ concentration decreased after stimulation with NS and NS together with TNF*α* (*P* < 0.0001 and *P* < 0.001, resp.) and after stimulation with ACA and ACA together with TNF*α* (*P* < 0.05 and *P* < 0.01, resp.). An inhibitor of MAP kinase, PD, alone had no effect on PGE_2_ secretion (Figure 4(b)).

### 3.5. Immunolocalization of TNF*α*/TNFRs Protein in Feline Endometrium

#### 3.5.1. Immunolocalization of TNF*α*

**Figure 5:**
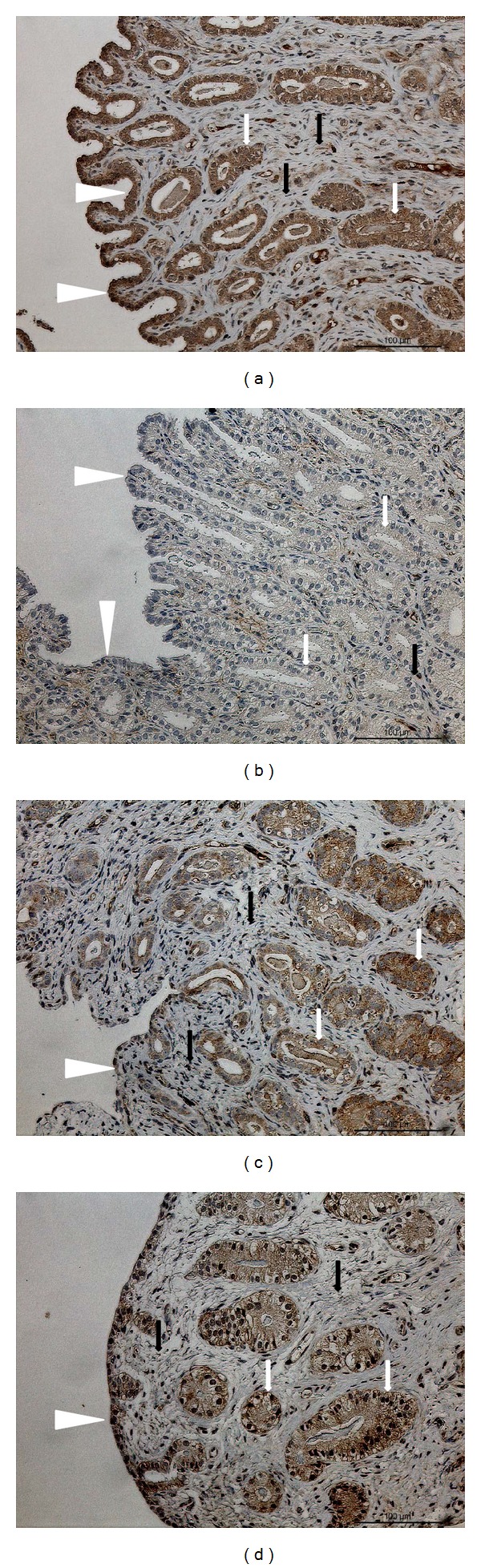
TNF*α* protein localization in endometria. Immunohistochemical (IHC) localization of TNF*α* in the endometria of cats collected at (a) estrus phase (E; *n* = 7) and (b) diestrus phase (D, *n* = 8) from (c) cats that had been treated with a P_4_ derivative analog, medroxyprogesterone acetate (MPA, *n* = 5; (d) cats presenting the clinical symptoms of pyometra (PYO, *n* = 3). (a) In females at estrus, strong TNF*α* signals are localized in glandular and surface epithelium; (b) in females at diestrus, some weak signals are localized in endometrial stroma; (c) in MPA females, TNF*α* expression is localized mostly in deep glands and weaker signals are present in surface epithelium; (d) in PYO females, signals are mostly localized in both the surface and the glandular epithelium. Surface epithelium: white arrowheads; glandular epithelium: white arrows; endometrial stroma: black arrows.

Very strong signals were observed in the surface and glandular epithelium but very weak signals were seen in endometrial stroma in cats from group E (Figure 5(a)). No or only weak signals were observed in both epithelia and stroma in cats from group D (Figure 5(b)). In uteri from cats receiving MPA, no or only weak signals were observed in surface epithelium, weak to moderate signals were seen mainly in deep endometrial glands, and weak to moderate staining was observed in endometrial stroma (Figure 5(c)). In inflamed uteri, strong signals were observed in endometrial glands, whereas in the surface epithelium the staining was weak to moderate. In the endometrial stroma in pyometric cats, there were no or only weak signals (Figure 5(d)).

#### 3.5.2. Immunolocalization of TNFR1

**Figure 6:**
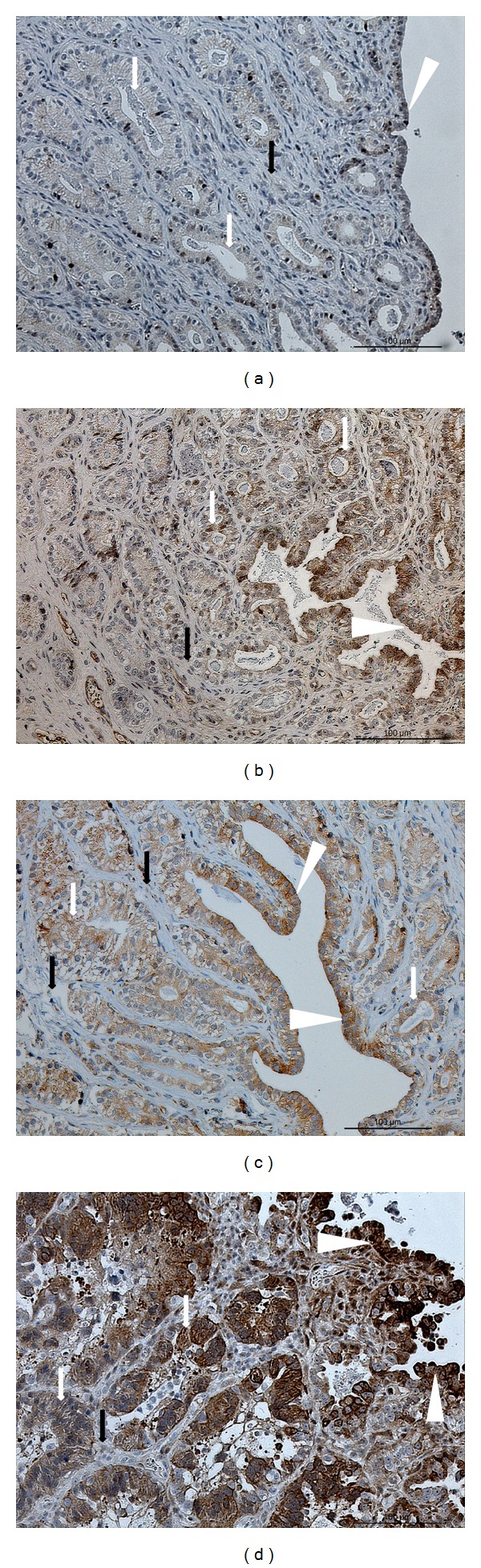
TNFR1 protein localization in endometria. Immunohistochemical (IHC) localization of TNFR1 in the endometria of cats collected at (a) estrus phase (E, *n* = 7) and (b) diestrus phase (D, *n* = 8) from (c) cats that had been treated with a P_4_ derivative analog, medroxyprogesterone acetate (MPA, *n* = 5); or (d) cats presenting the clinical symptoms of pyometra (PYO, *n* = 3). (a) In females at estrus, only weak TNFR1 signals are localized in the glandular and surface epithelium; (b) in females at diestrus, positive staining is localized in the glandular and surface epithelium, similar to (c) MPA females; (d) in PYO females, strong signals are mostly localized in both the surface and the glandular epithelium. Surface epithelium: white arrowheads; glandular epithelium: white arrows; endometrial stroma: black arrows.

Weak intensity scores were observed in the endometrial glands from group E or group D cats (Figures 6(a) and 6(b), resp.), whereas in the surface epithelium, staining was weak to moderate. The IHC analysis revealed abundant positive signals in surface and glandular epithelia from inflamed uteri (Figure 6(d)) and distinct diversification of staining in the surface epithelium of cats receiving MPA (Figure 6(c)). In that group, TNFR1 protein expression was weak to moderate in the endometrial glands. No or only weak signals were observed in endometrial stroma in all experimental groups.

#### 3.5.3. Immunolocalization of TNFR2

**Figure 7:**
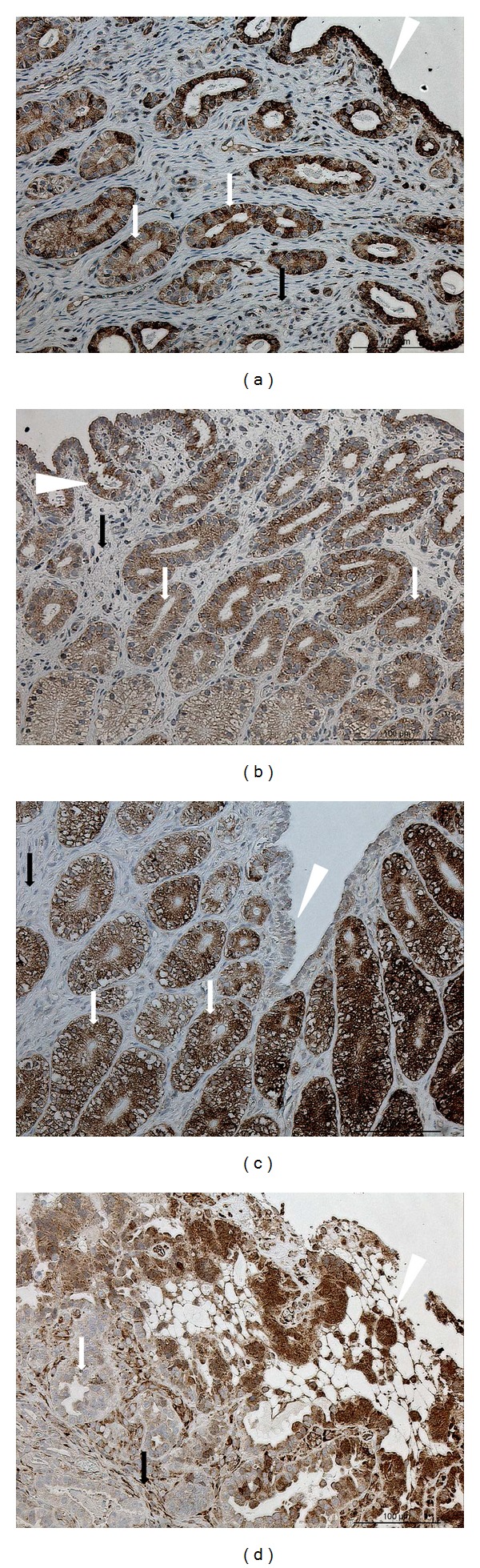
TNFR2 protein localization in endometria. Immunohistochemical (IHC) localization of TNFR2 in the endometria of cats collected at (a) estrus phase (E, *n* = 7) and (b) diestrus phase (D, *n* = 8) from (c) cats that had been treated with a P_4_ derivative analog, medroxyprogesterone acetate (MPA, *n* = 5); or (d) cats presenting the clinical symptoms of pyometra (PYO; *n* = 3). In females at (a) estrus and (b) diestrus, TNFR2 expression is localized in the glandular and surface epithelium; (c) in MPA females, very strong signals are observed in the endometrial glands, whereas (d) in PYO females, strong signals are present in surface and glandular epithelium and weaker ones in endometrial stroma. Surface epithelium: white arrowheads; glandular epithelium: white arrows; endometrial stroma: black arrows.

Moderate to strongly positive signals were localized in endometrial glands in all groups (Figures 7(a), 7(b), 7(c), and 7(d)). Similarly, moderate to strongly positive signals were observed in surface epithelium in all groups (Figures 7(a), 7(b), and 7(d)) with the exception of MPA-treated queens (Figure 7(c)), in which the staining levels were identified as none, weak, or moderate. No or only weak signals were observed for stromal TNFR2 protein expression in almost all tissue sections examined.

**Figure 8:**
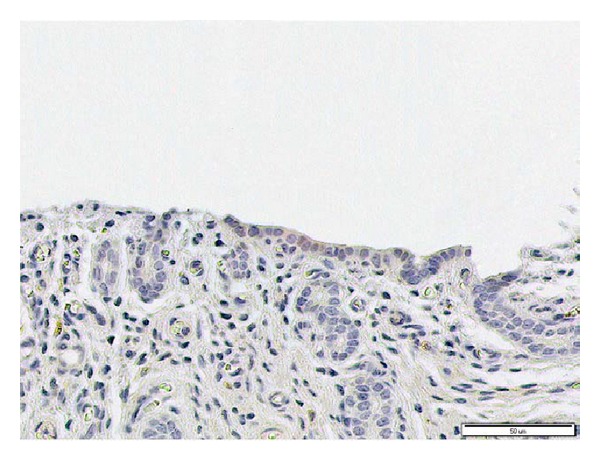
Isotype control with a nonimmunized rabbit serum at a dilution of 1 : 25. No positive signals are observed in the feline endometrium.

**Figure 9:**
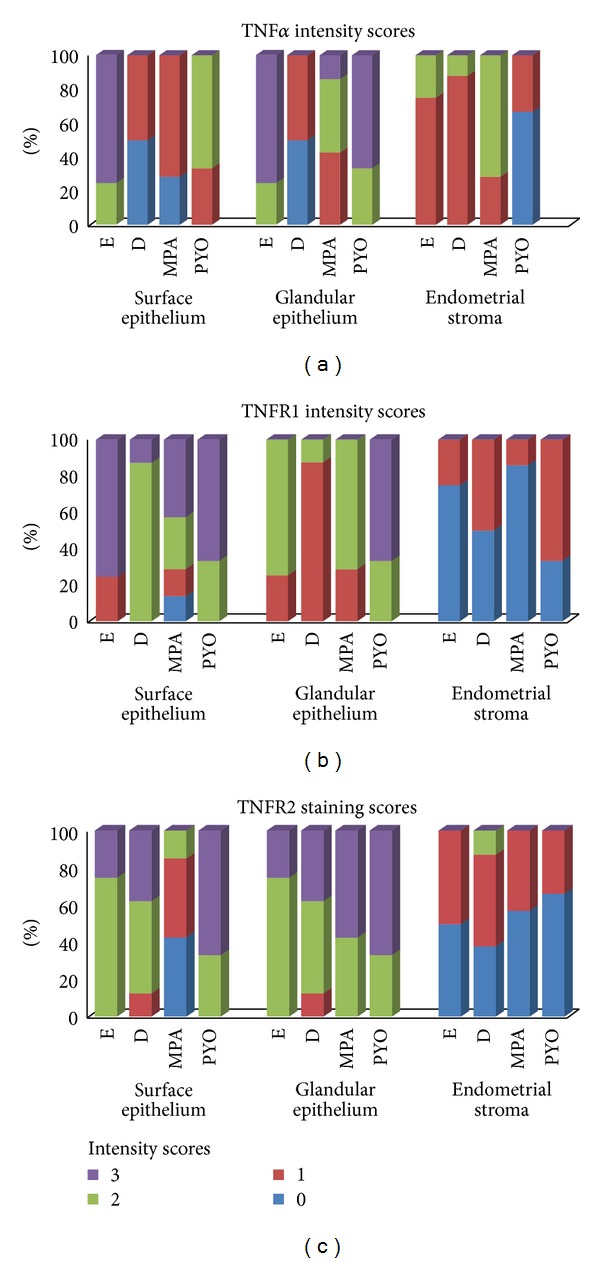
Graphic illustration of the relative intensity scores for TNF*α*, TNFR1, and TNFR2 in different compartments of the endometria of cats collected at estrus (E, *n* = 7) and diestrus (D, *n* = 8) from cats that had been treated with a P_4_ derivative analog, medroxyprogesterone acetate (MPA, *n* = 5), or cats presenting the clinical symptoms of pyometra (PYO, *n* = 3).

The negative (isotype) control showed no staining (Figure 8). The immunohistochemical scoring results are shown in Figure 9.

## 4. Discussion

In the present study, the separately cultured epithelial or stromal cells secreted TNF*α* following LPS stimulation. This observation stands in contrast to data obtained in bovine endometrial cells, in which there was no detectable level of TNF*α* after LPS challenge [25]. Similarly, in supernatants from isolated murine epithelial or endometrial stromal cells stimulated with various LPS, including LPS purified from* E. coli* O55:B5 as in the present study, TNF*α* concentrations were low [26]. These discrepancies may be due to species variations or to the use of different methods for TNF*α* detection. In the present study ELISpot was used instead of ELISA. ELISpot immunoassays are designed for the detection and enumeration of single cells secreting cytokines or other antigens. These assays are highly sensitive and allow identification of the secreting cells even when frequencies of these cells fall below 1 in 100,000 [27]. The authors of previous studies on bovine [25] and murine [26] endometrium concluded that the neighbouring cells, like macrophages, are more likely responsible than epithelial or stromal cells for LPS-challenged TNF*α* production. An increase in TNF*α* production followed by elevated PG secretion undoubtedly may have an important role in innate resistance of the endometrium to infection. Interestingly, in the present study the number of TNF*α* spots observed after LPS stimulation was distinctly higher in the endometrial epithelial cells than in stromal cells. Indeed, the epithelial cells regulate defensive strategies by orchestrating innate immune responses [28].

In our previous study, we showed that isolated fragments of feline uteri produce PGs after supplementation of the culture media with TNF*α* [19]; similar results were shown with endometrium of the cow [29], pig [30], horse [31], and human [32]. Prostaglandins are synthesized from AA by an enzymatic cascade and regulate a variety of processes in reproduction and immune function [33]. The primary enzyme responsible for inflammation-induced enzymatic liberation of AA from membrane phospholipids is PLA_2_. Consequently, inhibition of PLA_2_ should diminish PG synthesis. Previous studies showed that TNF*α*/TNFR1 complex activates PLA_2_ [34] and experiments with ACA abrogated TNF*α*-challenged PG synthesis in the bovine endometrial stroma [35]. In contrast to the earlier study [35], we clearly demonstrated that TNF*α* enhanced PG secretion in feline epithelial, not stromal cells. However, in accordance with the report by Skarzynski et al. [35], we noticed an inhibiting effect of ACA on TNF*α*-challenged PG synthesis in feline epithelium. In addition, a selective COX-2 inhibitor, NS, completely abolished TNF*α*-induced as well as basal PG synthesis. The latter effect stands in contrast to earlier results, in which COX-2 selective inhibitors, among them NS, minimally affected basal PGE_2_ secretion, although they were extremely effective in abolishing TNF*α*-stimulated PGE_2_ synthesis [36]. The present study confirmed that PLA_2_ and COX-2 are involved in TNF*α*-challenged PGs secretion in cat endometrial cells, since the enhancing effect of TNF*α* on PGs synthesis was abrogated by PLA_2_ or COX-2 inhibitors. Binding of TNF*α* with its receptor has been demonstrated to activate several signaling pathways, including MAPK [37]. Furthermore, an activation of MAPK is implicated as a signaling pathway for COX-2 gene transcription and protein expression, that is, in vascular smooth muscle cells [38]. Consequently, we expected that administration of PD 98059 should abolish TNF*α*-challenged PG secretion. Surprisingly, the enhancing effect of TNF*α* on PGE_2_ was not blocked after incubation of epithelial cells with MAPK inhibitor. In contrast, TNF*α* did not elevate PGF_2*α*_ secretion, when administered together with PD. In the study by Sakumoto et al. [39], MAPK inhibitor abolished TNF*α*-enhanced PG synthesis in bovine endometrium.

Tumor necrosis factor *α* acts through two distinct receptors, TNFR1 and TNFR2, although TNFR1 initiates the majority of the biological activities of TNF*α*. Activation of TNFR1 triggers an intracellular cascade that results in the initiation of two major transcription factors, nuclear factor *κ*B (NF-*κ*B) and c-Jun. These transcription factors are responsible for the inducible expression of genes important for various biological processes, including immune and inflammatory responses [37]. In the present study, TNF*α*/TNFR1 and 2 were shown to be differentially expressed depending on the uterine conditions. In cats in the luteal phase, TNF*α* immunolabeling was poor and restricted mostly to the stromal cells and, to a lesser extent, to the surface and glandular epithelium. In the group of cats receiving MPA, the TNF*α* immunostaining was stronger than in cats in luteal phase. The most distinct signals were observed in the superficial and glandular epithelium of the cats suffering from endometritis/pyometra complex. In one immunohistochemical study on TNF*α* in the canine endometrium during the course of the estrous cycle, positive signals were found in the endometrial stroma and in both superficial and glandular epithelium [40]. The intensity of the stromal TNF*α* staining was the highest in anestrus and proestrus dogs and diminished towards estrus and diestrus [40]. Neither superficial nor glandular epithelium showed statistical differences in TNF*α* immunoreactivity during the canine estrous cycle and TNF*α* immunostaining remained low to moderate in the course of the estrous cycle in dogs [40]. In the present study TNF*α* immunoreaction seemed to be similar in the surface and glandular epithelium in cats in estrus and was scored as moderate to strong; however, TNF*α* protein immunostaining seemed to be much less distinct in cats from diestrus. Immunostaining in the endometrial stroma appeared unaffected by stage of the estrous cycle, as similarly observed in the dog [40]. Immunolocalization of TNF*α* receptors varied depending on the receptor type. The most intense TNFR1 immunostaining was observed in the surface and glandular epithelium, with similar intensity in cats suffering from pyometra. Distinct TNFR1 immunostaining was also identified in animals receiving MPA especially in the surface and, to a lesser extent, in glandular epithelia. These observations emphasized that in the uteri of pyometric cats the pathological process affects mostly the epithelial compartment of the endometrium, not the stroma. Moderate or strong immunostaining for TNFR2 was observed in the epithelia of queens receiving MPA as well as those animals in estrus or diestrus, but it appeared less strong than in inflamed uteri. No signals or poor staining intensity was observed in the feline stroma for both receptors. This observation is in agreement with a previous study in cows, in which TNFR1 and TNFR2 proteins were weakly expressed in the stroma [41]. The sources of endometrial TNF*α* were the superficial and glandular epithelial cells and the endothelial lining of blood vessels located in the bovine endometrium [41]. That study showed significantly higher expression of TNF*α* in the luteal and follicular endometrium [41], suggesting its regulation by sex steroids. A similar result, with regard to the spatial localization of TNF*α* and TNFR1 protein, was obtained for equine endometrium; however, intensity of immunostaining was not affected by the phase of the estrous cycle [31]. Observations made in cows partially agreed with the present observation that the source of TNF*α* was the epithelium, not the stroma, at least in follicular endometrium [41]. In the present study, TNF*α* seemed to be more weakly expressed in the luteal phase endometrium compared with follicular phase endometrium, which is not exactly identical with our previous observations made with whole uterine fragments. In that study, uterine TNF*α* protein expression and mRNA transcription did not differ between cats in the follicular or luteal phases [19]. This discrepancy may be due to different methods used for TNF*α* analysis. Furthermore, the overriding goal of the present study was to measure semiquantitatively TNF*α* and its receptors in endometrium that was inflamed or exposed to exogenous P_4_ and to compare these observations with protein immunoreactivity in the follicular or luteal phases. The present observations confirmed that the TNF*α*/TNFRs complex is overexpressed in the inflammatory uterus and may be involved in the development of the changes in the endometrial glands of cats receiving exogenous P_4_ as a hormonal contraceptive.

In summary, this study shows that feline epithelial endometrial cells produce TNF*α* as a result of LPS stimulation, which emphasizes the defensive role of epithelial cells during infections with Gram-negative bacteria. Tumor necrosis factor *α*-augmented PG secretion is abolished by PLA_2_ and COX-2 and, partially, by MAPK inhibitors. Changes in TNF*α*/TNFR1 and 2 expression were discernable in the uteri of cats receiving octane medroxyprogesterone compared with those of estrus and diestrus queens, so this medication may favor the development of endometritis and pyometra in cats.
